# 单细胞RNA测序技术在肺癌肿瘤微环境研究中的进展

**DOI:** 10.3779/j.issn.1009-3419.2024.101.15

**Published:** 2024-06-20

**Authors:** Yanhong WANG, Bin LUO, Zhuo WANG, Zujun QUE, Lei JIANG, Jianhui TIAN

**Affiliations:** ^1^200071 上海，上海中医药大学附属市中医医院肿瘤临床医学中心（王衍鸿，罗斌，阙祖俊，田建辉）; ^1^Clinical Oncology Center, Shanghai Municipal Hospital of Traditional Chinese Medicine, Shanghai University of Traditional Chinese Medicine, Shanghai 200071, China; ^2^200071 上海，上海中医药大学附属市中医医院肿瘤研究所（王衍鸿，罗斌，阙祖俊，田建辉）; ^2^Institute of Oncology, Shanghai Municipal Hospital of Traditional Chinese Medicine, Shanghai University of Traditional Chinese Medicine, Shanghai 200071, China; ^3^200032 上海，复旦大学生物医学研究院（王卓）; ^3^Institute of Biomedical Research, Fudan University, Shanghai 200032, China; ^4^200433 上海，同济大学附属上海市肺科医院（蒋雷）; ^4^Pulmonary Hospital, Tongji University, Shanghai 200433, China

**Keywords:** 单细胞RNA测序, 肺肿瘤, 免疫治疗, 肿瘤异质性, 肿瘤微环境, Single-cell RNA sequencing, Lung neoplasms, Immunotherapy, Tumor heterogeneity, Tumor microenvironment

## Abstract

免疫微环境对肿瘤的发生发展起着关键作用。近年来，随着高通量测序技术的飞速发展，研究人员对肿瘤微环境中的免疫细胞组成及其功能有了更深入的了解。然而，传统的群体测序技术难以解析单个细胞层面的异质性，限制了对肿瘤微环境复杂性的全面理解。单细胞RNA测序技术的兴起，为揭示肺癌免疫微环境的异质性带来了新的机遇。当前以T细胞为中心的免疫治疗在临床中容易出现免疫原性耐药或者免疫相关性肺炎等影响预后的副作用，其关键因素是肿瘤微环境中免疫细胞与肿瘤细胞的相互作用发生了变化。而单细胞RNA测序技术可以从细胞间互作、拟时序分析等角度揭示肿瘤微环境中不同亚群间的起源与作用，进而发现新的细胞亚群或新生生物标志物，为揭示免疫治疗的耐药及疗效监测等提供新的途径。该综述系统回顾了单细胞RNA测序技术在揭示肺癌特别是免疫治疗后肺癌微环境异质性方面的最新研究进展，为促进肺癌免疫治疗的精准化与个体化提供参考。

## Abstract (en)

The immune microenvironment plays a key role in the development and progression of tumors. In recent years, with the rapid advancement of high-throughput sequencing technologies, researchers have gained a deeper understanding of the composition and function of immune cells in the tumor microenvironment. However, traditional bulk sequencing technologies are limited in resolving heterogeneity at the single-cell level, constraining a comprehensive understanding of the complexity of the tumor microenvironment. The advent of single-cell RNA sequencing technology has brought new opportunities to uncover the heterogeneity of the immune microenvironment in lung cancer. Currently, T-cell-centered immunotherapy in clinical settings is prone to side effects affecting prognosis, such as immunogenic drug resistance or immune-related pneumonia, with the key factor being changes in the interactions between immune cells and tumor cells in the tumor microenvironment. Single-cell RNA sequencing technology can reveal the origins and functions of different subgroups within the tumor microenvironment from perspectives such as intercellular interactions and pseudotime analysis, thereby discovering new cell subgroups or novel biomarkers, providing new avenues for uncovering resistance to immunotherapy and monitoring therapeutic efficacy. This review comprehensively discusses the newest research techniques and advancements in single-cell RNA sequencing technology for unveiling the heterogeneity of the tumor microenvironment after lung cancer immunotherapy, offering insights for enhancing the precision and personalization of immunotherapy.

目前，免疫检查点抑制剂（immune checkpoint inhibitors, ICIs）的出现推动了免疫治疗的飞速发展^[1]^。现在应用于临床的ICIs主要有针对程序性死亡受体/配体-1（programmed cell death 1/ligand 1, PD-1/L1）的帕博利珠单抗/阿替利珠单抗^[2]^、针对细胞毒性T淋巴细胞抗原-4（cytotoxic T-lymphocyte antigen 4, CTLA-4）的伊匹单抗^[3]^等。这些药物的应用为肺癌的治疗带来了一场革命，成为肿瘤治疗领域的新里程碑^[4]^。然而，肺癌是一种高度异质性的肿瘤，不仅存在肿瘤细胞的异质性，还存在肿瘤细胞异质性导致的微环境异质性^[5,6]^。研究^[7]^表明，肿瘤微环境（tumor microenvironment, TME）的异质性对肿瘤的进展和对ICIs的反应具有重要影响，是肿瘤治疗面临的卡脖子难题之一。因此，有必要对肺癌生态系统进行全面的研究。

单细胞RNA测序（single-cell RNA sequencing, scRNA-seq）是一种对单个细胞水平上的转录组进行测序的技术。该技术的特点是能够识别由单细胞基因组突变引起的差异基因表达，并鉴定出新的细胞特异性标记和细胞类型。通过scRNA-seq技术，我们不仅可以更深入地了解TME中细胞亚群的分布及状态，还能够从全新的角度解析TME中细胞间的相互作用。在肺癌的研究中，scRNA-seq技术除了能够揭示调节肺癌免疫应答的关键因素，还能寻找并预测免疫治疗可能的靶点和潜在预测疗效的新型分子标志物，为提高免疫治疗的效率和精准性提供重要线索，见图1。

**图1:**
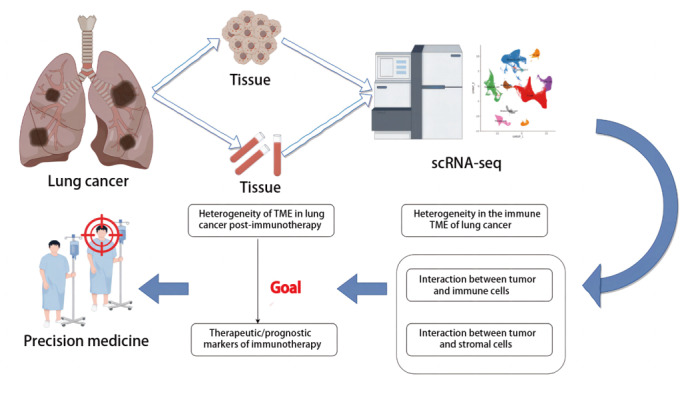
单细胞RNA测序技术研究肺癌免疫治疗后TME

## 1 scRNA-seq技术揭示肺癌TME的异质性

自2009年汤富酬等^[8]^首次完成scRNA-seq技术以来，这一方法因其在研究细胞异质性方面的卓越能力而在科学界受到广泛关注。目前，基于细胞分选技术的差异，在scRNA-seq中两种最常见的方法是基于液滴的scRNA-seq和基于微孔的scRNA-seq。基于液滴的scRNA-seq技术平台，包括10X Genomics Chromium、InDrop和Drop-seq^[9]^。该技术的优点是低试剂和样品体积以及短细胞加载时间。10X Genomics因其高吞吐量和稳定性在临床研究中广泛使用，Drop-seq因成本低廉、易于设置而受到青睐，而InDrop则提供灵活的样本处理选项，适合定制研究。相较之下，基于微孔的scRNA-seq技术如BD Rhapsody、SeekOne、M20 Genomics和Singleron Matrix等，尽管在吞吐量和成本上有优势，但由于商业化仪器认可度不高或操作协议较复杂，这些平台并未被广泛采用。

TME构成了肿瘤细胞和其周边组织及基质细胞之间的复杂相互作用网络，这些相互作用不仅对肿瘤生长有着显著影响，也关系到肿瘤的侵袭扩散及患者预后。由于肿瘤细胞间的异质性，特别是在肺癌中，这进一步加剧了免疫治疗的难度。

为了克服这一难题，scRNA-seq技术已被广泛应用在肺癌的研究当中。该技术能够在细胞层面上解析TME的异质性，发现新的肿瘤、免疫和基质细胞亚群。这些新亚群可能具有不同的生物学特征和临床表现，为肺癌的个体化治疗提供了可能。范国平研究团队^[10]^运用scRNA-seq技术针对表皮生长因子受体（epidermal growth factor receptor, EGFR）突变的早期肺腺癌（lung adenocarcinoma, LUAD）组织和癌旁正常肺组织细胞的全基因转录谱开展研究。该研究发现了TM4SF1^+^/SCGB3A2^+^恶性肿瘤细胞亚群，这些细胞在受到浸润免疫细胞所分泌的IL-1B等炎性细胞因子的刺激时，ELF3基因被上调，从而激活PI3K/Akt/NF-κB通路，上调与增殖和抗凋亡相关的肿瘤基因表达。2020年5月，Hae-Ock Lee和Myung-Ju Ahn的团队^[11]^对44例肺癌患者的肿瘤样本进行了scRNA-seq。这项研究不仅发现了一种与肺癌转移密切相关的癌细胞亚型tS2，还揭示了非小细胞肺癌（non-small cell lung cancer, NSCLC）患者中肿瘤来源的血管内皮细胞亚群的重构降低了其抗原呈递和免疫细胞的归巢活性，为理解肺癌TME中的细胞行为和肿瘤免疫逃逸机制提供了新的见解。Yuen等^[12]^使用scRNA-seq技术发现接受抗PD-L1治疗无效的患者中存在高表达IL-8的髓系细胞的亚群，该细胞亚群与抗原呈递机制的下调有关，并进一步指出血浆IL-8的表达水平可能成为预测ICIs治疗效果的生物标志物。在发现基质细胞亚群方面，在Mathieson等^[13]^的研究中，发现了一种特定的肿瘤相关成纤维细胞（cancer-associated fibroblast, CAF）亚群FAP^+^、PDPN^+^、αSMA^-^CAF-S5。尽管它们位于肿瘤区域的较远位置，但CAF-S5的存在与NSCLC患者的较差生存预后相关。

基于这些发现，我们能更深入地理解肺癌的生物学机制，并为开发针对特定亚群的治疗策略提供生物信息学依据。2018年，Bernard Thienpont领导的研究团队^[14]^成功创建了首个完整的肺癌细胞图谱。该图谱包含52个不同的基质细胞亚群和12种癌细胞亚群，其中不仅包括肺癌细胞，还涵盖了TME中的内皮细胞、免疫细胞和成纤维细胞等非肿瘤细胞。其中CD8^+^ T细胞亚群表达较高的免疫检查点分子水平，除了经典免疫靶点PDCD1和CTLA4外，还包括目前临床试验中的后起之秀LAG3、TIGIT、HAVCR2/TIM3、CD27和TNFRSF9/CD137等，为识别和开发新的免疫治疗靶点提供了强有力的科学基础^[15]^。周彩存团队^[6]^绘制了晚期NSCLC的肺癌细胞及其TME的细胞类型特异性转录组图谱，揭示了肿瘤异质性与肿瘤相关中性粒细胞的相关性，为中性粒细胞在NSCLC及免疫治疗中的潜在功能研究提供理论依据。这些研究均提示我们，应用scRNA-seq技术能够精确地识别和描绘TME中的细胞亚群以及其基因的特异性表达的情况。这不仅能为诊断和预后提供参考，还可以辅助药物靶点的发现和新型治疗策略的研发^[4,16]^，对精准医疗特别是免疫治疗的意义重大。

## 2 scRNA-seq技术揭示TME中细胞间的相互作用及功能串扰

scRNA-seq技术对于分析肺癌中的免疫细胞在TME中的作用显示出巨大的潜力。利用CellPhoneDB、CellChat等细胞交互分析算法可以研究细胞与细胞之间的相互关系，从而揭示肿瘤细胞与免疫、基质细胞之间的复杂相互作用。对细胞相互作用进行分析有助于我们更深入地理解肿瘤生物学，为优化治疗策略提供新的可能性。

在这一研究领域，Trajanoski教授^[17]^整理了19项研究的scRNA-seq数据（包括298例患者的505个单细胞测序样本），发现组织驻留中性粒细胞（tissue-resident neutrophils, TRNs）在NSCLC中的多样性和可塑性。特别是，通过CellPhoneDB分析，发现NSCLC细胞与TRNs之间的KDR-VEGFA信号轴显著上调，这一发现揭示了该信号轴在TME中可能的免疫抑制作用。同时，韩昱晨团队利用scRNA-seq技术对不同阶段LUAD的单细胞转录组研究发现FOLR2^+ ^TAM与NR4A3^+^ CD4^+ ^T通过诱发树突状细胞（dendritic cells, DC）分泌趋化因子CCL17/19/22到TME中，进而招募CD4^+^ T细胞^[18]^。该研究提出“FOLR2^+^ TAM/NR4A3^+^ CD4^+^ T/Treg轴”在LUAD的演进中发挥潜在作用，为理解LUAD中免疫抑制性微环境的形成提供了新的见解，并为针对巨噬细胞和T细胞亚群的癌症治疗干预策略开辟了新的研究方向。四川大学华西医院李为民教授团队^[19]^通过对NSCLC患者的肿瘤样本和相匹配的临近组织进行scRNA-seq分析，发现了一个新巨噬细胞（Mφ）亚群--SELENOP-Mφ。他不仅可以诱导上皮-间充质转化（epithelial-mesenchymal transition, EMT），塑造TME，促进肿瘤侵袭性，增强Treg细胞反应和肿瘤免疫力，还与淋巴细胞的功能密切相关。

除了研究免疫细胞外，通过scRNA-seq技术，我们还可以识别各种基质细胞的特征、异质性表达谱^[20]^，并进一步探究它们在肿瘤免疫抑制过程中的作用机制，从而更深入地理解肿瘤免疫反应的复杂性和多样性^[21]^。肿瘤基质主要由CAF、血管内皮细胞以及细胞外基质（extracellular matrix, ECM）等构成^[22]^。Hanley等^[23]^在NSCLC中识别出三种成纤维细胞亚群：周围性成纤维细胞、肺泡性成纤维细胞和肌成纤维细胞。其中肌成纤维细胞在肿瘤组织中高度富集，通过与上皮分化丧失、TP53突变和髓系细胞等途径影响TME，与较差的生存率密切相关。另一项scRNA-seq研究^[24]^发现了一簇高度表达ECM蛋白基因的CAFs，这簇细胞能通过细胞间交流增加调节性T细胞（regulatory T cells, Treg）中PD-L1和CTLA-4蛋白的水平，从而引发免疫治疗的耐药性。此外，基于scRNA-seq技术进行的研究^[25]^还发现NSCLC患者的血管内皮细胞通过重塑过程可减少其抗原呈递和抑制免疫细胞的归巢活性，从而有助于肿瘤的免疫耐受。在LUAD中，通过scRNA-seq技术发现，FAP^+^ PDPN^+^ CAF亚群和ACTA2^+^ MCAM^+^外周血管细胞亚群通过NOTCH信号通路与肿瘤细胞相互作用^[26]^。具体来说，这些基质细胞接收来自内皮细胞的NOTCH信号，激活NOTCH3通路，从而增强胶原蛋白的产生并促进肿瘤细胞的侵袭。这种基质细胞与肿瘤细胞的相互作用推动了TME的重塑，并且可能影响肿瘤的免疫抑制和治疗抵抗。

在免疫治疗研究中，利用scRNA-seq技术分析肺癌TME内的免疫细胞和基质细胞，有助于深入理解肺癌及其TME的异质性和多样性，为我们揭示肿瘤对ICIs治疗的耐药机制提供了可能。

## 3 scRNA-seq技术揭示免疫治疗后肺癌微环境的异质性

正如前文所述，scRNA-seq技术的应用不仅可以用来研究肺癌原发的TME，还可以用来揭示免疫治疗后肺癌微环境的异质性，使研究人员能够研究免疫治疗对不同细胞亚群的具体影响以及TME的重塑情况。scRNA-seq技术已经在揭示免疫治疗后肺癌TME的异质性方面取得了重大进展。通过对不同研究团队的成果进行综合分析，我们可以看到这项技术如何使科学家能够深入了解肺癌免疫治疗后对免疫细胞亚群的变化情况。

首先，利用scRNA-seq技术可以分析免疫治疗对各亚群功能变化的影响。张泽民教授团队^[27]^利用scRNA-seq技术，深入探究接受PD-1抑制剂治疗后的NSCLC患者外周血T细胞的功能变化情况，研究结果显示，肿瘤相关CD4^+ ^T细胞亚群展现出了比CD8^+ ^T细胞亚群更高的细胞毒活性。其次，利用scRNA-seq技术分析免疫治疗下的免疫细胞亚群变化，可协助发现新的免疫标志物。张鹏课题组^[28]^通过scRNA-seq技术研究了NSCLC患者接受新辅助PD-1阻断联合化疗后的TME变化，结果发现显著病理缓解（major pathologic response, MPR）的患者中富含FCRL4^+ ^FCRL5^+^记忆B细胞和CD16^+ ^CX3CR1^+^单核细胞的转录特征。与此同时，衰老的CCL3^+^中性粒细胞会通过正反馈回路与SPP1^+^ TAM相互作用，从而导致免疫治疗反应不佳。这些发现不仅有助于探索免疫预后标志物方面，还为理解治疗后免疫细胞的变化提供了深入见解，并为克服免疫治疗抵抗提供了潜在策略。

周彩存教授团队^[6]^还通过scRNA-seq分析了TME中不同细胞类型之间的相互作用，以全面了解NSCLC患者的TME，预测免疫治疗后的疗效。他们的研究覆盖了血管生成、T细胞激活、CAF激活、免疫抑制细胞招募以及检查点途径的激活等方面。通过对不同细胞间互作分子的分析，研究人员发现了一个涉及多种致癌和炎症信号传导途径的复杂网络，特别是巨噬细胞在通过检查点途径抑制T细胞方面起了关键作用。研究还发现，不同的NSCLC亚型在主要信号途径的激活上也存在差异。LUAD中TIGIT通路高度激活，而TIM3通路激活较低。此外，除了极少数的肺鳞状细胞癌（lung squamous cell carcinoma, LUSC）患者外，PD-1/PD-L1轴并未表现出显著激活，这可能与PD-1/PD-L1在转录组水平上的低表达有关。

最后，该技术还能用来对罕见病例进行深入分析以开发新的生物标志物以提高免疫治疗的有效性。通常情况下，表达PD-L1阴性的NSCLC患者不会从帕博利珠单抗治疗中获益。然而，上海市胸科医院的韩宝惠团队^[29]^利用scRNA-seq技术，发现了受益于帕博利珠单抗治疗的PD-L1阴性NSCLC患者的外周血单个核细胞（peripheral blood mononuclear cell, PBMC）中，NKG7^+^ NK和NKG7^+^ T（NK-T）细胞亚群显著减少，而未成熟的T细胞和CD8^+^ T细胞亚群则增加。此外，该研究还识别了ID2、PIK3CD、UQCR10、MATK、MZB1、IL7R和TRGC2等分子标记物，可以用于预测PD-1/PD-L1治疗的效果。

通过以上这些实例，我们可以看到scRNA-seq技术如何揭示PD-1/PD-L1等ICIs治疗后肺癌TME的复杂性和异质性，为克服免疫治疗抵抗、理解治疗后免疫细胞的变化以及开发新的治疗策略提供了宝贵的见解。这些研究不仅促进了对肺癌免疫反应的深入理解，而且为进一步优化肺癌的免疫治疗策略提供了科学依据。

## 4 单细胞空间组学技术揭示免疫治疗后肺癌微环境的异质性

肿瘤的细胞空间分布是不均匀的，其中肿瘤亚克隆的分布和免疫微环境的空间异质性是导致大多数癌症种类异质性和免疫治疗反应差异的关键因素^[30]^。单细胞分辨率下的空间组学技术结合了基于图像的空间蛋白质组学技术以及DNA、RNA变异分析，能够在细胞结构的背景下定量检测众多基因和蛋白质，从而提供宝贵的分子、细胞和微环境信息^[31]^。这使研究者能够从空间结构角度探讨细胞间相互作用、肿瘤细胞与TME的相互关系，以及患者对免疫治疗的反应^[32]^。

Zugazagoitia教授^[33]^对接受PD-1抑制剂治疗的晚期NSCLC患者进行了空间转录组学分析，发现CD56在免疫细胞中高表达并且与患者免疫治疗的获益相关。Moutafi等^[34]^的研究表明，NSCLC细胞中CD44的高表达与PD-1抑制剂治疗后患者生存期的延长有关，可以作为独立的预后指标。同时，研究还揭示了CD44高表达的肿瘤细胞促进特定免疫微环境的形成，并上调如PD-L1和TIM-3等免疫调节分子的表达。这些发现支持CD44作为一个新的生物标志物，对于优化肺癌患者的免疫治疗策略具有潜在价值。

## 5 单细胞多组学技术揭示肺癌免疫微环境异质性

近年来，单细胞蛋白组学、基因组学、表观基因组学和T细胞受体（T cell receptor, TCR）分析等多种单细胞技术，也在肺癌TME异质性研究中取得了显著进展。单细胞蛋白组学技术如质谱流式细胞技术（cytometry by time of flight, CyTOF）和成像质谱细胞术（imaging mass cytometry, IMC）不仅提供了肺癌TME中免疫细胞的详细地图^[35]^，还可用于分类和定位浸润免疫细胞，指导免疫治疗的选择和实施^[36]^。单细胞基因组测序技术可以揭示肺癌亚型和肿瘤进展的异质性^[37]^，而单细胞表观基因组学则能揭示染色质动态和调控机制，为了解基因表达改变提供了新视角^[38]^。单细胞多组学分析整合不同单细胞组学数据集，加深了我们对肺肿瘤中免疫细胞异质性和作用的理解，为免疫疗法研究提供了新的靶点^[39]^。特别是单细胞TCR分析对理解T细胞反应的微妙变化至关重要，TCR测序可阐明T细胞激活、选择和分化的途径，进而有助于改善肺癌免疫疗法的疗效和精准度^[40]^。这种综合应用的单细胞多组学技术不仅拓宽了我们对于肺癌免疫微环境中细胞异质性和动态变化的理解，而且还促进了对于肺癌发生、发展及对治疗反应机制的深刻洞察。特别是，这些技术在揭示免疫细胞的复杂网络以及在肿瘤抵抗中的作用方面，提供了前所未有的细节，从而为免疫治疗策略的优化开辟了新途径。

## 6 scRNA-seq技术在肺癌领域的临床应用

进一步而言，正是基于TME的复杂性，scRNA-seq技术在研究肺癌中浸润免疫细胞的应用才逐渐得到重视。通过深入分析肺癌TME内部的细胞异质性和免疫细胞的动态变化，该技术能够助力我们深入理解肺癌患者固有免疫与适应性免疫的功能状态。这些功能状态不仅在消灭肺癌细胞的过程中发挥着关键作用，同时也对于开发有效的免疫治疗方案、识别潜在的治疗靶标以及预测患者预后至关重要（表1）。scRNA-seq技术的应用，也有助于揭示免疫治疗失败的潜在机制，将显著提升肺癌免疫治疗的效果和患者的生存预后。

**表1:** 单细胞测序技术在肺癌免疫微环境研究中的临床发现

Author	Research technique	Type of lung cancer	Cell type	Main finding
Tian et al.^[5]^	10x Genomics ®scRNA-seq	SCLC	Tumor cell, T cell, Mac	In SCLC, patients with low immune features are more likely to benefit from ICB therapies than those with strong immune features. The study established a detailed immune atlas for SCLC and classified T cells, revealing patterns of dysfunction and exhaustion markers like PDCD1, CTLA4, HAVCR2, LAG3, TIGIT and LAYN. These markers are potential targets for immunotherapy. Additionally, non-neuroendocrine SCLC subtypes show increased interactions with other cells, including immune and stromal cells, which may correlate with clinical outcomes of immunotherapy.
Lavin et al.^[35]^	10x Genomics ®scRNA-seq, CyTOF	LUAD	Tumor cell, T cell, Mac	The study identified characteristic genes of tumor-infiltrating macrophages such as TREM2, CD81, MARCO and APOE. It also detailed the immune cell landscape in early-stage LUAD, highlighting a significant increase in Tregs and enhanced PD-1 expression in CD4^+^ and CD8^+^ T cells during early tumor stages. These findings deepen the understanding of the immune microenvironment in LUAD and suggest potential targets for future immunotherapy strategies.
Sun et al.^[56]^	VITApilote® snRandom-seq, IMC	LUAD	B cell, plasma cell, T cell	An extensive single-cell analysis revealed distinctive immune niches in different organ-specific environments. Notably, primary LUAD and adrenal gland metastases exhibited a significant presence of B cells, plasma cells, and various T cell subtypes (including activated, exhausted, and memory T cells), suggesting a robust immune environment conducive to immunotherapy effectiveness. Conversely, immunosuppressive environments characterized by the presence of collagen I and high expression of the T cell exhaustion marker TIM-3 were identified in brain and liver metastases, indicating a potential challenge for immunotherapy in these sites.
Sorin et al.^[57]^	10x Genomics ®scRNA-seq, IMC	NSCLC	Tumor cell, T cell, monocyte	The study found that CXCL13 expression is associated with the efficacy of ICIs. Recombinant CXCL13 can enhance the in vivo response to anti-PD-1 therapy, possibly due to the increase in antigen-stimulated T cell subgroups and the reduction in CCR2^+^ monocytes.
Leader et al.^[58]^	10x Genomics ®scRNA-seq, CITE-seq, TCR-seq	NSCLC	Tumor cell, T cell, Plasma cell, Mac	The study developed a detailed immunological profile of early-stage lung cancer and introduced the LCAM module consisting of PDCD1^+ ^CXCL13^+^ activated T cells, IgG^+^ plasma cells, and SPP1^+^ macrophages, which serves as a direct indicator of antigen-specific anti-tumor immune activation. This framework assists in understanding and enhancing immunotherapeutic strategies.
Zhu et al.^[59]^	10x Genomics ®scRNA-seq, ST	LUAD	Tumor cell, Treg cell	The study identified a UBE2C^+^ subpopulation in LUAD crucial for tumor invasiveness. In the LUAD TME, NK cells and MALT^+^ B cells increased significantly in early stages, whereas Treg cells and secretory B cells decreased as LUAD progressed to advanced stages. These findings support the development of personalized treatment strategies for aggressive LUAD.
Sinjab et al.^[60]^	10x Genomics ®scRNA-seq, ST	LUAD	Tumor cell, Dendritic cell, Mac	This study identified distinct immune checkpoint receptor and cytokine receptor interactions within different regions of LUAD. Decreased overlap of L-R interactions was observed in distant tumor regions compared to proximal areas. Additionally, increased interactions involving immune checkpoint proteins CD24 with LGALS9 in tumor epithelial cells, and SIGLEC10 in dendritic and macrophage cells, as well as HAVCR2, suggest that regional differences in these interactions could affect the tumor's immune microenvironment and impact the effectiveness of immunotherapy.
Zhang et al.^[61]^	10x Genomics ®scRNA-seq	LUAD	Tumor cell, CD4^+^/ CD8^+^ T cell, NK cell, Treg cell, M2- Mac	Malignant cells with low R-loop scores exhibit glycolysis and epithelial-mesenchymal transition pathway activation and immune escape promotion. R-loop score correlates with T cell exhaustion and immune evasion, predicting therapeutic response to targeted therapy, chemotherapy, or immunotherapy. FANCI-mediated R-loop changes affect Ras signaling, inhibiting tumor proliferation and dissemination.

SCLC: small cell lung cancer; LUAD: lung adenocarcinoma; ICB: immune checkpoint blockade; Mac: Macrophage; snRandom-seq: nucleus RNA sequencing technology; IMC: imaging mass cytometry; CyTOF: cytometry by time-of-flight; Treg cell: T regulatory cell; ICIs: immune checkpoint inhibitors; NSCLC: non-small cell lung cancer; CITE-seq: cellular indexing of transcriptomes and epitopes sequencing; TCR-seq: T cell receptor sequencing; ST: spatial transcriptomics; TME: tumor microenvironment.

在筛选免疫治疗的预后标志物方面，scRNA-seq技术的应用尤为重要。通过对单个细胞的基因表达进行精确测量，研究人员能够识别出与肿瘤生存率、治疗响应和免疫治疗效果密切相关的关键基因和细胞亚群。例如，通过分析LUSC患者的scRNA-seq数据，研究发现编码葡萄糖转运蛋白-1（glucose transporter 1, GLUT1）的溶质载体家族2成员1（solute carrier family 2 member 1, SLC2A1）在肿瘤组织中表达上调^[41]^，并且其编码的蛋白质GLUT1与患者的生存率呈负相关，为LUSC患者的治疗选择和个性化免疫治疗策略提供了重要信息。中国医学科学院肿瘤医院的研究人员通过分析LUAD患者的单细胞转录组，揭示了肺癌TME中的免疫细胞异质性和细胞间交互^[42]^。通过分析这些scRNA-seq数据，研究建立了一个整合的11个基因（包括HLA-DPB1、FAM83A、ITGB4等）的预后标志物模型。同时，作者还在晚期患者中，发现HLA-DRB6^+^巨噬细胞亚群表现出更活跃的炎症反应，并显示出与肿瘤细胞的相互作用增强，这表明它们在疾病进展中的潜在作用。这有助于预测肺癌患者的生存预后及免疫治疗的响应。北京协和医院的马文斌团队^[43]^通过scRNA-seq技术，鉴定了肺癌原发和转移标本中免疫细胞分布的差异，并识别出与脑转移（brain metastasis, BM）相关的上皮细胞亚群--BMAECs，并使用机器学习算法开发了代表高BM风险的BM-index。

在理解肿瘤免疫微环境的复杂性方面，天津医科大学肿瘤医院任秀宝与尤健教授的研究团队^[44]^利用scRNA-seq技术分析了IIIA期接受NSCLC帕博利珠单抗联合化疗新辅助有疗效响应的患者与无响应患者的标本，发现了决定疗效的几个主要免疫学事件，包括肿瘤病灶内更多的三级淋巴结构形成、B细胞和CD4^+ ^T细胞的协同增加、B细胞向抗肿瘤IgG类别转换以及治疗前外周血TCR多样性和CD8^+ ^T细胞的克隆扩增，均能提高抗肿瘤免疫反应。此外，还发现肿瘤组织内富集的LAMP3^+^DC亚群通过多种配体受体相互作用（如CCL22/CCR4、CCL17/CCR4和CCL19/CCR7）与CD4^+ ^T、CD8^+ ^T和B细胞进行互作，促进淋巴细胞的募集和活化。此外，LAMP3^+ ^DCs还通过IL-15与浆细胞互作，支持B细胞的增殖和分化。本研究为新辅助免疫联合化疗临床反应中协同相互作用的细胞机制提供了新见解，并提供了潜在的预测性生物标志物和治疗靶点，以改善积极的临床结果。

在个性化治疗策略的制定方面，复旦大学附属中山医院胸外科卢春来教授团队^[45]^发现LUAD组织的UPP1高表达与患者的不良预后相关。UPP1通过转化生长因子-β1（transforming growth factor-β1, TGF-β1）的高分泌以及PD-L1的上调共同促进LUAD免疫抑制微环境的构建。UPP1^high^的肿瘤细胞亚群对博苏替尼、达沙替尼等靶向药物更敏感。抑制UPP1可增加CD8^+^ T细胞的细胞毒性并增敏PD-L1单抗免疫治疗效果。该研究发现UPP1可以作为LUAD潜在的治疗靶点，对UPP1的抑制可能能够增敏PD-L1/PD-1免疫治疗的效果。在接受ICIs治疗的NSCLC患者中，张勇等^[46]^发现间质表皮转化因子（mesenchymal to epithelial transition factor, MET）基因高表达的患者对治疗不敏感，研究人员通过scRNA-seq技术对超过20,000个免疫细胞进行了深入分析，在MET基因高表达的患者中识别出一种新的XTIST^+^/CD96^+^/KLRG1^+^的NK细胞亚群。在免疫治疗产生抵抗的患者中，这一NK细胞亚群的比例明显升高，而NK细胞和CD8^+^ T细胞亚群的比例则出现下降。基于这一现象，研究人员提出了一种新的治疗策略，即联合使用MET抑制剂与ICIs，以增强抗肿瘤免疫，帮助肿瘤消退。

综上所述，scRNA-seq技术在肺癌治疗领域的应用展示了其在识别新的预后标志物、理解肿瘤免疫微环境的复杂性，以及为个性化治疗策略的制定提供科学依据方面的重要价值。

## 7 scRNA-seq技术的局限性

scRNA-seq在运用中逐步显露出一些本质的方法学问题。首先，scRNA-seq分析中mRNA的捕获效率仅介于5%-15%，这一局限性带来了数据的稀疏性、采样偏误以及低表达基因信息的遗失^[47,48]^。其次，该技术只适用于新鲜的组织样本，而对于未经解离预处理的冷冻临床样本，scRNA-seq便无法施行。这不仅缩小了其应用领域，也提升了实验的复杂度，同时减少了可分析的样本数量^[49,50]^。第三，细胞解离的过程本身也会诱导应激基因的表达，引发细胞转录模式发生“人工改变”，继而产生转录偏差。这样获得的数据无法真实地反映样本细胞的转录状况，严重影响了实验结果的可信度。例如，Brink等^[51]^研究发现，37 ^o^C下的蛋白酶分离步骤会诱发应激基因表达，引入实验误差，导致细胞类型鉴定不准确。对比实验进一步证实了这种限制，即37 ^o^C下的细胞分离会导致多个应激基因表达增加，使得结果严重扭曲，而低温分离则可以有效规避这一问题^[52]^。第四，在肺脏等固态组织中，蛋白酶倾向于分离易于操作的细胞，忽略难以分离的细胞。同时，过度的解离操作可能损害肺脏中的敏感细胞^[53]^。因而，可能不能全面捕获肺脏所有细胞类型，影响研究结果的准确性。第五是在肺癌研究中，scRNA-seq可能会出现“双细胞”现象，这是因为在细胞解离过程中，一滴液体内可能含有两个或多个携带相同条形码的细胞，导致这些细胞被错误地作为单个细胞进行计数。这些双细胞分为两类：同源双细胞，即源自同一细胞类型；异源双细胞，来自不同转录活性细胞，造成人工杂交的转录组。异源双细胞对下游分析的影响更为显著，包括降维、细胞聚类、差异表达分析和细胞发育轨迹等。尽管降低加载细胞浓度可以有效控制双细胞的数量，但这也意味着可分析的细胞总数减少，成本显著上升。为了解决这些问题，研究者们开发了各种检测双细胞的计算方法，例如Chord、Doublet Finder等^[54,55]^，以提高数据分析的准确性和稳定性。最后，收集肺癌患者免疫治疗前后的标本也面临多重临床挑战。研究需要获取同一患者手术前未治疗的活检样本及接受新辅助免疫治疗后的术后样本，这在实际操作中极具挑战。由于新辅助免疫治疗自2020年起才逐步推广，相关病理数量较少，这限制了相关研究的进行。此外，这一过程中涉及的伦理和技术问题也增加了收集样本的难度。为了研究需要，研究者可能会不自觉地将不需要新辅助免疫治疗的患者纳入治疗计划，对患者造成过度医疗，从而引发了伦理上的争议。其次，确保免疫治疗前后样本的质量和完整性对于后续的单细胞测序至关重要，但两次取样的时间跨度和临床操作的复杂性往往难以保证这一点，从而影响了肺癌免疫治疗后scRNA-seq研究的真实性和可靠性。尽管存在这些挑战，随着技术的持续进步和突破，scRNA-seq技术在肺癌研究与免疫治疗方面的应用前景仍然被看好。

## 8 小结与展望

scRNA-seq技术彻底改变了我们对肺癌的认识方式，它为肿瘤细胞和TME的异质性研究提供了前所未有的洞察。这些技术不仅加深了我们对肺癌的认识，还促进了对肺癌免疫治疗反应的理解，更为免疫治疗策略的发展指明了方向。通过单细胞水平上对肺癌异质性的深入研究，研究人员可以识别出导致免疫治疗抗性的细胞亚群，揭示肿瘤-免疫相互作用特征，发现新的生物标志物，理解免疫治疗耐药性背后的机制，以及鉴定并靶向干预肿瘤驻留的免疫细胞。此外，scRNA-seq技术还可应用于患者液体活检的分析，实现对治疗反应和疾病进展的非侵入性监测，从而实时调整治疗方案，推进精准医疗。
